# A Prospective Profile of Visual Field Loss following Stroke: Prevalence, Type, Rehabilitation, and Outcome

**DOI:** 10.1155/2013/719096

**Published:** 2013-09-09

**Authors:** Fiona J. Rowe, David Wright, Darren Brand, Carole Jackson, Shirley Harrison, Tallat Maan, Claire Scott, Linda Vogwell, Sarah Peel, Nicola Akerman, Caroline Dodridge, Claire Howard, Tracey Shipman, Una Sperring, Sonia MacDiarmid, Cicely Freeman

**Affiliations:** ^1^Department of Health Services Research, Whelan Building, University of Liverpool, Brownlow Hill, Liverpool L69 3GB, UK; ^2^Altnagelvin Hospitals HHS Trust, Altnagelvin BT47 6SB, UK; ^3^NHS Ayrshire and Arran, Ayr KA6 6DX, UK; ^4^Royal United Hospitals Bath NHS Trust, Bath BA1 3NG, UK; ^5^Fairfield Hospital, Bury BL9 7TD, UK; ^6^Durham and Darlington Hospitals NHS Foundation Trust, Durham DH1 5TW, UK; ^7^Ipswich Hospital NHS Trust, Ipswich IP4 5PD, UK; ^8^Gloucestershire Hospitals NHS Foundation Trust, Gloucester GL1 3NN, UK; ^9^St Helier General Hospital, Jersey JE1 3QS, UK; ^10^University Hospital NHS Trust, Nottingham BG7 2UH, UK; ^11^Oxford Radcliffe Hospitals NHS Trust, Oxford OX3 9DU, UK; ^12^Salford Royal NHS Foundation Trust, Salford M6 8HD, UK; ^13^Sheffield Teaching Hospitals NHS Foundation Trust, Sheffield S10 2JF, UK; ^14^Swindon and Marlborough NHS Trust, Swindon SN3 6BB, UK; ^15^Wrightington, Wigan and Leigh NHS Trust, Wigan WN1 2NN, UK; ^16^Worcestershire Acute Hospitals NHS Trust, Worcester WR5 1DD, UK

## Abstract

*Aims*. To profile site of stroke/cerebrovascular accident, type and extent of field loss, treatment options, and outcome. *Methods*. Prospective multicentre cohort trial. Standardised referral and investigation protocol of visual parameters. *Results*. 915 patients were recruited with a mean age of 69 years (SD 14). 479 patients (52%) had visual field loss. 51 patients (10%) had no visual symptoms. Almost half of symptomatic patients (*n* = 226) complained only of visual field loss: almost half (*n* = 226) also had reading difficulty, blurred vision, diplopia, and perceptual difficulties. 31% (*n* = 151) had visual field loss as their only visual impairment: 69% (*n* = 328) had low vision, eye movement deficits, or visual perceptual difficulties. Occipital and parietal lobe strokes most commonly caused visual field loss. Treatment options included visual search training, visual awareness, typoscopes, substitutive prisms, low vision aids, refraction, and occlusive patches. At followup 15 patients (7.5%) had full recovery, 78 (39%) had improvement, and 104 (52%) had no recovery. Two patients (1%) had further decline of visual field. Patients with visual field loss had lower quality of life scores than stroke patients without visual impairment. *Conclusions*. Stroke survivors with visual field loss require assessment to accurately define type and extent of loss, diagnose coexistent visual impairments, and offer targeted treatment.

## 1. Introduction

Stroke or cerebrovascular accident is estimated to occur in approximately 150,000 people per year in the UK, and disabilities following stroke affect about 300,000 [1]. Visual field loss is reported as occurring in 8–67% [2–7] although some visual field impairment is due to a previous stroke or preexistent ocular pathology [7]. Estimates vary widely as the proportion testing positive is highly dependent on the time after stroke. Visual field loss is a loss of part of the field of vision which may occur centrally or peripherally. However, following stroke, loss of visual field is more usually peripheral in nature. The most common type of visual field loss is that of homonymous hemianopia in which there is loss of the same half of the visual field in both eyes and which occurs in approximately two-thirds of those with visual field loss [7–9]. Other types of visual field loss may include inferior and superior quadrantanopia, constricted visual fields, scotomas, and altitudinal defects [10–15].

Visual field loss following stroke has largely been attributed to cortical strokes in which the visual pathway is damaged. The intracranial course of the anterior visual pathway includes the optic nerves which pass medially from the optic canals to form the optic chiasm and are supplied by branches of blood vessels from the ophthalmic artery and pial vessels from adjacent branches of the internal carotid artery [16–19]. The optic chiasm is formed by the mergence of the two optic nerves and receives its blood supply from an anastomosis of arterioles from the Circle of Willis [20–22].

The posterior visual pathway extends from the optic chiasm through to the visual cortex. The optic tracts sweep laterally from the optic chiasm, passing around the ventral portion of the midbrain and encircling the hypothalamus posteriorly. The optic tracts obtain their blood supply via a pial plexus which is continuous anteriorly with that of the optic chiasm and fed partly from the posterior communicating artery and branches of the middle cerebral artery but mainly from the anterior choroidal artery [23]. The lateral geniculate body is located in the diencephalon and has a dual blood supply involving the anterior choroidal artery and lateral choroidal artery [23]. 

The optic radiations consist of superior, inferior, and central nerve fibre bundles. The superior and central bundles pass directly posteriorly through the posterior temporal and parietal lobes. The inferior bundle initially passes anteriorly to loop into the temporal lobe before passing posteriorly through the parietal lobe. The blood supply to the optic radiations is predominantly from the posterior and middle cerebral arteries [24]. The nerve fibres of the optic radiations terminate in the visual striate cortex (V1) which is located on the medial aspect of the occipital lobe, superior and inferior to the calcarine fissure. The cortex is supplied predominantly by the posterior cerebral artery and its calcarine branch. A parieto-occipital branch supplies the superior calcarine lip, a posterior temporal branch supplies its inferior lip, and a calcarine branch supplies the central region posteriorly. The middle cerebral artery may supply the posterior aspect of the calcarine sulcus with an anastomosis between posterior and middle cerebral arteries accounting for sparing of the macula in cases of posterior cerebral artery occlusion [24, 25]. 

Pambakian and Kennard [26] reported visual field loss due to occipital lobe lesion in 40%, parietal lobe in 30%, temporal lobe in 25%, and 5% with damage to optic tract and lateral geniculate nucleus. Zhang et al. [10] reported the area of stroke as occipital lobe in 54%, optic radiations in 33%, tract in 6%, lateral geniculate nucleus in 1%, and 5% with multiple pathway segment involvement. Further reports state that most stroke related visual field loss related to occipital infarct [27–29].

Homonymous hemianopia on admission is linked to poor early survival and conversely around 10% [30] experience full spontaneous recovery within the first 2 weeks. Visual field defects seriously impact on functional ability and quality of life following stroke [31]. Patients with visual field defects have an increased risk of falling, impaired ability to read, poor mood, and higher levels of institutionalisation [32–36]. Visual field loss impacts on a patient's ability to participate in rehabilitation, may ultimately result in poor long term recovery, and can lead to loss of independence, social isolation, and depression [4].

National guidelines in the UK recommend that every patient with stroke has a practical assessment of vision and examination of visual field [37, 38] with access to appropriate therapy. Treatment for visual field loss includes restitution, substitution, and compensatory options [39]. Compensatory options, in particular, have shown favourable effects on improved visual scanning into the hemianopic side [40, 41].

Previous studies on visual field loss following stroke have provided information on type of visual field loss, treatment options, or recovery in both clinical and experimental settings. The Vision In Stroke (VIS) study is a prospective observation study aimed at capturing data on types of visual impairment following stroke and to report the profile of those visual impairments in standard care clinical environments. One objective of this study is to prospectively evaluate the prevalence of visual field loss occurring in this prospective clinical population of stroke survivors with suspected visual impairment. This paper provides a review of the visual pathway and, from the clinical population, profiles visual field loss in terms of type and extent of visual field loss, site of causative lesion, the treatment options, and outcome.

## 2. Methods

### 2.1. Study Design and Population

The design of this study is a prospective multicentre observational case cohort study. The Vision In Stroke (VIS) group consists of local investigators from twenty UK hospital trusts who are responsible for assessing stroke patients and collecting patient data. The data is collated centrally at the University of Liverpool. The study has multicentre ethical approval via the National Research Ethics Service and is undertaken in accordance with the Tenets of Helsinki. The recruitment period for this study ran from May 2006 to April 2009 with followup to April 2010.

The target population was stroke patients suspected of having a visual difficulty. Referrals could be made from in-patient wards, rehabilitation units, community services, or out-patient clinics. Patients were given an information sheet and recruited after informed, written consent. Patients were excluded if they were unable to consent due to cognitive impairment, unwilling to consent, if their diagnosis was that of transient ischaemic attack, or if they were discharged without vision assessment.

### 2.2. Measures

Patients with suspected visual difficulty were identified using a screening form. Subsequently this was used as the referral form to the orthoptic service. A standardised investigation sheet was used for the eye assessment consisting of identification of known preexistent ocular pathology, symptoms and signs, investigation of visual field, ocular motility, and perceptual aspects. Visual fields were assessed qualitatively by traditional confrontation methods or quantitatively by Humphrey (Humphrey systems, Dublin, CA, USA) automated central and/or peripheral static perimetry or Goldmann/Octopus (Haag Streit Int, Switzerland) kinetic perimetry. Complete hemianopia was defined as macular splitting field loss involving all of the superior and inferior quadrants to one side of the visual field. Partial hemianopia was defined as macular sparing, incongruous, and/or partial hemifield involvement. 

Visual acuity was assessed at near and distance fixation with Snellen or logMAR acuity tests. Low visual acuity was considered in two categories. The first defined low visual acuity as less than best corrected 6/12 Snellens acuity or 0.3 logMAR in accordance with UK driving standards [42]. The second defined low visual acuity as less than 6/18 Snellens acuity or 0.5 logMAR and equal or better than 3/60 Snellens acuity as per World Health Organization (WHO) guidelines [43]. 

Assessment of ocular alignment and motility consisted of cover test, evaluation of saccadic, smooth pursuit and vergence eye movements, retinal correspondence (Bagolini glasses), fusional vergence (20D or fusional range), stereopsis (Frisby near test), prism cover test, and lid and pupil function. 

Perceptual deficits were recorded after questioning of the patient and/or carers and relatives. Inattention was assessed by means of a combination of assessments including line bisection, Albert's test, cancellation tests, memory tests using verbal description, and drawing. Alexia was diagnosed where patients described an inability to read (despite being able to see the text) because of being unable to decipher the words or their meaning or being unable to make sense of the text. 

Quality of life was undertaken using the Activities of Daily Living Dependent on Vision (ADLDV) questionnaire [44]. This consists of 22 questions related to vision including visual recognition, personal care and hygiene, mobility, and reading. It uses a Likert scale of 1–4 indicating the individual cannot see to do through to having no difficulty. A full “normal” score is 88.

Stroke details were recorded from patient notes accounting for stroke laterality, type, and area involved. Ocular treatment details were recorded along with outcome. Reasons for nonattendance at review appointments included death, a move out of area, lost to followup, followup unwanted, or unknown.

### 2.3. Data Analysis

Results were inputted to the statistical package SPSS version 19 (IBM SPSS Statistics, USA). Pearson chi squared test (*χ* ^2^) was undertaken to analyse cross tabulations of results for visual field loss and outcome of followup versus factors such as age, presence of other visual impairment, laterality and area of stroke, and recovery. Multiple regression analysis was undertaken for analysis of quality of life scores versus age at stroke onset, side and type of stroke, gender, and duration from stroke onset to time of first eye examination. A *t* test was used to analyse differences between similar measurements with normal distributions including laterality of visual field loss.

## 3. Results

### 3.1. General Demographics

**Figure 1:**
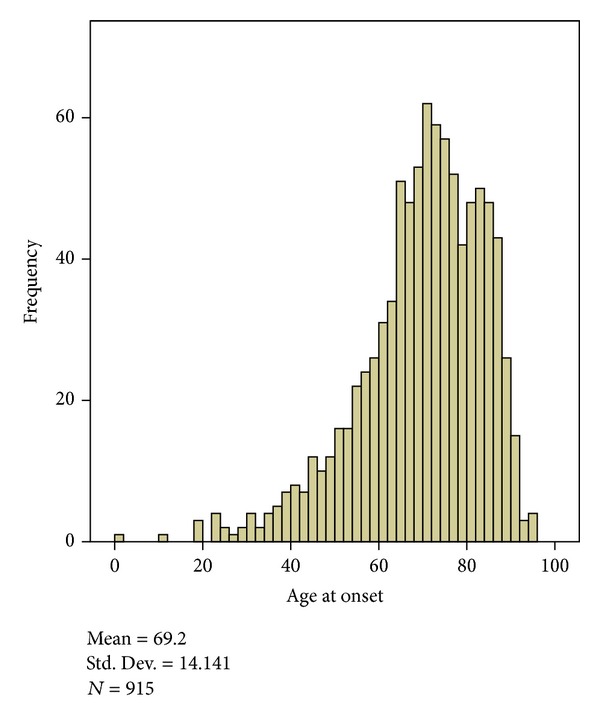
Frequency indicates the numbers of patients with age of onset of stroke.

1345 patients were referred for visual assessment for this study. 915 patients were recruited, and 430 patients were excluded, the latter mainly due to inability to provide informed, written consent as required of the ethical approval for this study (Table 1). Of 915 patients recruited, 59% (*n* = 540) were male and 41% (*n* = 375) female. Mean age at onset of stroke was 69 years (range 1–94: SD 14 years, Figure 1). One patient was aged 1 year and the range thereafter was 19 to 94 years. The median age at onset of stroke was 71 years. 

Median duration from onset of stroke to initial baseline eye examination was 22 days (0–2543 days), the mean of 40.84 (SD 141.28) days being skewed by three outliers who were referred a number of years after the stroke onset. Stroke lesion was right sided in 448 patients (49%, i.e., right-sided brain), left sided in 348 (38%), and bilateral in 119 (13%). Infarcts accounted for 773 cases (84.5%) with the remainder due to haemorrhage.

### 3.2. Visual Field Loss

Ethical approval allowed the documentation of visual diagnosis (if available) for excluded patients but not the documentation of full visual data. Of 430 excluded patients, 336 had outline visual data. Of these, 164 (48.8%) had visual field loss recorded and mainly of homonymous hemianopia type. 479 (52.3%) of 915 recruited patients had visual field loss. Thus, 51.4% of all patients referred to the VIS study (both recruited and nonrecruited patients) had visual field loss.

**Figure 2:**
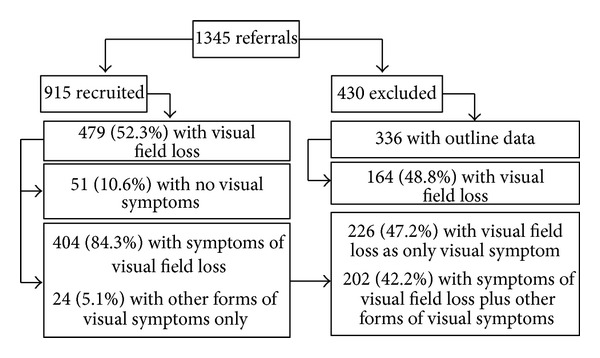
Visual symptoms.

404 (84%) of the 479 recruited patients with visual field impairment complained of the symptom of visual field loss. Fifty-one patients (10.6% of 479 patients) had no visual symptoms. Visual field loss was the sole visual symptom in 226 patients (47.2%). 202 patients (42.2%) complained of additional visual symptoms including reading difficulty, blurred vision, diplopia, and visual perceptual abnormalities (hallucinations, spatial difficulties, impaired colour vision, and alexia) (Figure 2). Visual field loss was right sided in 182 patients (38%), left sided in 256 patients (53.5%), and bilateral in 41 (8.5%). Preexistent visual field loss was noted in 30 patients (4.9%) accounting for the greater finding of left-sided visual field loss than the finding of right sided brain stroke. 

Assessment of visual field loss was by confrontation methods in 63% (*n* = 302) and quantitative assessment in the remainder including automated static central and peripheral perimetry (*n* = 129, 27%) and kinetic Goldmann perimetry (*n* = 48, 10%).

The most common type of visual field loss was found to be complete (*n* = 259, 54%) and partial (*n* = 93, 19.5%) homonymous hemianopia and occurring significantly more frequently to the left side than to the right side or bilaterally, *P* = 0.001 (*t* test). Other types (Table 2) included superior or inferior quadrantanopia (*n* = 73, 15.2%), temporal crescent defect (*n* = 1, 0.2%), constricted visual fields (*n* = 44, 9.2%), scotomas (*n* = 24, 5.1%), and bilateral hemianopia (cortical blindness: *n* = 8, 1.7%). 

### 3.3. Ocular History

Ocular pathology was noted in 17 patients (3.5%) with visual field loss including glaucoma (*n* = 9), retinal pathology (*n* = 5), and cataract (*n* = 3). Visual field loss in these patients was deemed to relate to their ocular pathology: the type was of constriction in all but one patient who had scotomatous loss (glaucoma patient). Thirteen patients (1.4%) had preexisting visual field loss due to a previous stroke. 

### 3.4. Area of Stroke

The area of brain involved by the stroke and producing visual field loss was most commonly the occipital and/or parietal lobes (*P* = 0.001: *χ* ^2^ test). Other affected areas reported by neuroimaging included periventricular and intraventricular lesions, cerebellum, brainstem, thalamus, basal ganglia, external and internal capsule, and temporal and frontal lobes in addition to lacunar strokes and infarcts of the anterior, middle, and posterior cerebral arteries and partial anterior circulation infarcts (Table 3). 

The types of visual field loss were compared for area of stroke lesion (Table 4). Homonymous hemianopia was prevalent in occipital lobe lesions and with middle and posterior cerebral artery infarcts. Homonymous quadrantanopia and constricted visual field loss were prevalent in parietal and temporal lobe lesions plus middle and posterior cerebral artery infarcts. Homonymous scotomas, altitudinal defects, and temporal crescent defects were all associated with occipital lobe lesions.

### 3.5. Associated Visual Impairment

151 patients (31%) had visual field loss as their sole visual impairment. In addition to a diagnosis of visual field loss, 328 patients had additional visual impairments. 28.6% (*n* = 137) of those with visual field loss also had low vision less than 0.3 logMAR, 28.2% (*n* = 135) had coexistent eye movement abnormalities, and 25% (*n* = 120) had visual perceptual difficulties (most being visual inattention: *n* = 93, 19.4%).

### 3.6. Visual Rehabilitation

Treatment was provided for 474 patients. No options for restitution treatment were provided. Compensatory options largely constituted advice on adaptive strategies using visual search exercises and visual field awareness for 250 patients (52.7%) or typoscopes (*n* = 43, 9%). Substitutive options included the use of substitutive prisms (*n* = 28, 6%): Table 5. In addition, treatment was targeted at relieving visual symptoms relating to coexistent visual impairment of eye movement or low vision using prisms, occlusion, and orthoptic exercises plus refraction and low vision aids. 

### 3.7. Outcome

**Figure 3:**
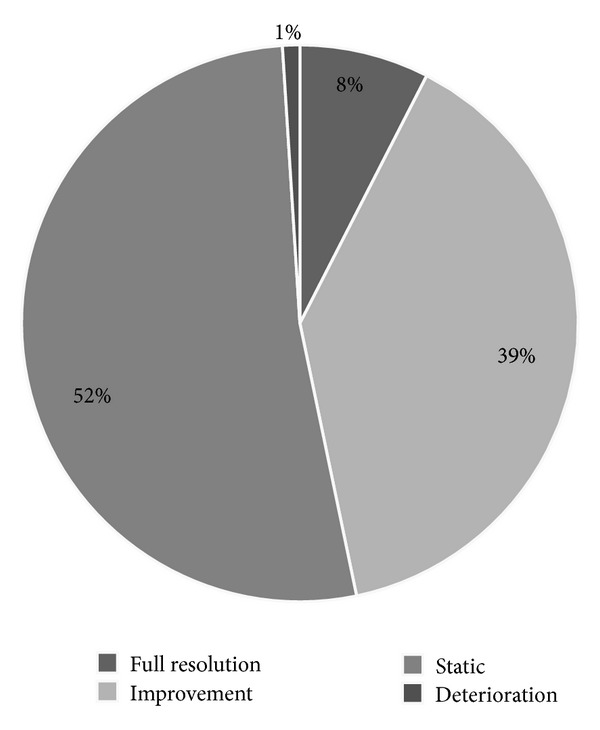
Outcome of visual field loss after followup.

Following baseline assessment and diagnosis of visual field loss, 64 patients were discharged from eye care services. 56 patients were referred to other eye care services, and 359 were offered review appointments. Table 1 also contains information on reasons for exclusion from this study. 199 patients attended their follow-up appointments which ranged from 2 weeks to 3 months postbaseline assessment (Figure 3). 

Fifteen patients (7.5%) had a full recovery of field loss. 78 patients (39.2%) showed partial improvement of visual field, and two patients (1%) showed further loss of visual field. 104 patients had stable, unchanged visual field loss (52.3%). There was no identifiable factor found to be associated with those patients achieving full restoration of their visual field, partial restoration, or no improvement regardless of their age at stroke onset (*P* = 0.058), gender (*P* = 0.075), area of stroke lesion (*P* = 0.222), or type of stroke (*P* = 0.869: *χ* ^2^ test). 

### 3.8. Quality of Life

**Figure 4:**
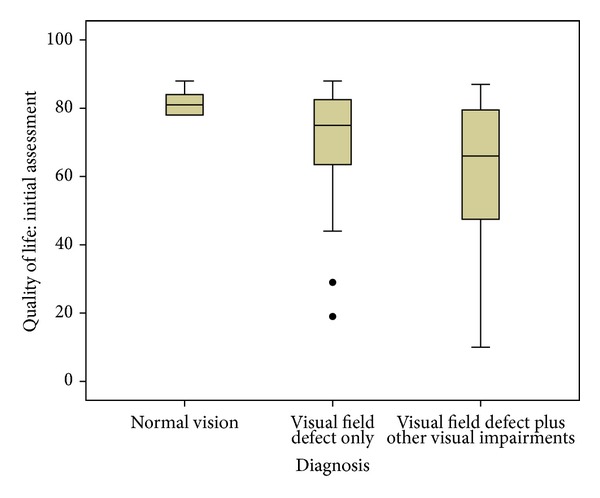
Quality of life measured with Activity of Daily Living Dependent on Vision questionnaire (maximum score of 88 indicating no impact).

ADLDV responses were obtained from 63 patients with visual field loss at their first appointment. The overall mean score was 65.69 (SD 18.07), compared to a possible maximum score of 88. For those patients with visual field loss as their only visual difficulty, their mean score was 69.43 (SD 18.89). For those patients with visual field loss plus additional visual impairments, the mean score was 63.55 (SD 17.47). Despite a slightly lower mean score for those with combined visual impairments, there was no significant difference (*P* = 0.988: *χ* ^2^) to those with solely visual field loss (Figure 4). In contrast, for patients from the overall stroke cohort who had no visual impairment when assessed at baseline, quality of life score was a mean of 81.66 (SD 3.88). 

Using a multiple linear regression model we analysed the relationship between the initial quality of life score and predictor variables of age at stroke onset, length of time from stroke onset to time of first eye examination, laterality, and type of stroke plus visual diagnosis. We obtained an overall *R* value of 0.267 (*F* = 0.676, *P* = 0.669). Regression coefficient 95% confidence intervals and the *t*-statistic were used to analyse the statistical significance of the model's coefficients for difference in relation to gender (CI: −17.76–5.33, *P* = 0.285), age at onset (CI: −3.8–0.52, *P* = 0.763), duration from onset to eye examination (CI: −0.3–0.19, *P* = 0.158), laterality (CI: −8.8–7.03, *P* = 0.823), type (CI: −21.45–15.51, *P* = 0.749), and visual diagnosis (CI: −4.76–2.84, *P* = 0.613). We obtained an *R* ^2^ value of 0.071.

## 4. Discussion

Visual field loss is reported in the acute period following stroke in 45–67% of patients [2, 30, 45, 46] and in the long term for 8–25% of patients following adjustment for recovery of visual field and morbidity [3, 47, 48]. 51.4% of all referred patients had demonstrable visual field loss with a prevalence of 52.3% for those formally recruited to the VIS study. A very small percentage of visual field defects were preexistent because of a previous stroke (1.4%) or because of coexistent ocular pathology (3.5%). It is important to ascertain the presence of preexisting visual field loss—whether stroke related or ocular related—so that visual field loss can be attributed to the recent stroke. Almost two thirds of patients had visual field assessment by confrontation methods with the remainder being assessed using quantitative perimetry methods. Confrontation has been found to be reliable on admission but less so for followup where there is some recovery [49]. However, Townend and colleagues [48] reported that hemianopia is likely to be underestimated by confrontation and automated perimetry is more sensitive. In view of partial and peripheral visual field defects, quantitative visual field assessment that tests peripheral and not just central visual field is also preferable. Furthermore, visual field loss should be measured by a quantifiable method which thus allows comparison of change over time [7, 9]. Kinetic perimetry using a moving target and static perimetry using on-off stationary targets may be undertaken. Where evaluation of field of vision for driving is required, a quantitative perimetry assessment using an Esterman test should be undertaken [50]. It is usual practice to assess right and left eyes separately. However, where quantitative perimetry is requested but the patient is unable to perform perimetry in either eye separately, we found that binocular assessment with the Esterman test or III4e Goldmann target is a useful screening assessment for homonymous visual field loss. Furthermore, it must be noted that not all acute-stage stroke survivors are able to physically undertake formal visual field perimetry assessments, and confrontation remains an essential assessment tool for such patients.

A greater number of our patients had left-sided visual field loss. A slightly greater preponderance for left-sided visual field loss has also been reported in one previous large cohort study [10]. The type of visual field loss was predominantly homonymous hemianopia which occurred as complete or partial loss (with or without macular sparing) in 348 patients (73.5%). Other types of visual field loss included inferior and superior quadrantanopia, constricted visual fields, scotomas, and altitudinal defects. Our findings were similar to those reported in the literature [10–15]. Zhang et al. [10] found that scotomas and macular sparing could not be localised to occipital lesions but could occur in other locations of the visual pathway: a statement with which we concur. 

Most of our patients (*n* = 404, 84%) complained of their visual field loss as a visual symptom. Others complained of reading difficulty, blurred vision, or visual hallucinations. Additional perceptual symptoms included spatial difficulties, alexia, and impaired colour vision. It should be noted that visual hallucinations are often unreported and it is likely that the prevalence of visual hallucinations following stroke related visual field loss is thus underestimated because patients avoid reporting this symptom fearing their cognitive state will be questioned [51, 52]. Previous reports have stated that reading difficulty occurs more commonly in right-sided than left-sided hemianopia. We have recently reported no difference in frequency of reading difficulty regardless of the laterality of the visual field loss [53], and in fact, we found left-sided hemianopia to be more common that right sided. 

Notably, 51 patients (10%) did not complain of any visual field loss and seemed unaware or unaffected by this deficit in their daily lives. Previous reports have also identified that a number of patients are unaware of their visual field loss but who also continue to drive. Thus there is an impact to driving and road safety [5, 54]. In such asymptomatic cases, additional objective observations must be considered to try to identify those with visual field loss, such as noting increased collisions and bumps to the affected side, exaggerated head movements, and turning of the head to view objects in the affected side. 

The brain imaging reports for our patients depicted either the area of stroke lesion, for example, occipital lobe, or the artery affected, for example, middle cerebral artery infarct. We noted multiple areas of the brain affected by stroke lesions in which visual field loss was documented. Typically we found cortical strokes were associated with visual field loss and particularly occipital, temporal, and parietal lobes plus middle and posterior cerebral artery infarcts. Unexpectedly, we also noted some stroke lesions to be reported in brainstem and cerebellar areas which are not associated with the visual pathway. In these cases, where previous stroke-related visual field loss was excluded, it has been assumed that the visual pathway was affected by an extension of the stroke that was not documented in the imaging report. 

When considering the type of field loss and location of stroke lesion, homonymous hemianopia was more prevalent in occipital lobe and middle or posterior cerebral artery strokes. Quadrantic defects were more prevalent in occipital, parietal, and temporal lobe strokes. Homonymous scotomas, altitudinal defects, and temporal crescent defects were associated with occipital lobe strokes. Thus, we recommend that postchiasmatic visual field loss should be screened for in patients with strokes affecting occipital, parietal, and temporal lobes or middle and posterior circulation strokes. It is equally important to remember that anterior circulation strokes may also impact on the visual pathway causing homonymous defects but also unilateral visual field defects with retinal stroke or optic nerve damage. 

There are three main approaches to visual rehabilitation: substitution, adaptation, or restitution [39]. A comparison of these indicated that greatest improvement in function follows visual search training [39]. Visual restorative therapy aims to restore the visual field, and treatment options may include flicker stimulation of the blind field which produces changes in cortical function with cortical reorganisation [55]. Improvement has been reported in locating moving flickering objects in the blind field, improved navigation skills, reading ability, and visual sensitivity [56, 57]. This type of treatment is not offered in any of our NHS centres and therefore was not a treatment available to our patients. However, adaptive and substitutive options were available. 

The profile of visual rehabilitation offered to our patients was largely dependent on the individual needs of these patients, regardless of age or presence of cognitive or communication problems. Compensatory options aim to adapt for the visual field loss by altering the patient's behaviour or their activity through exercises, training, and cues. In our study, treatment most frequently consisted of advice on adaptive visual search strategies. This encompassed improving awareness of the visual field loss and employing visual search strategies to promote the individual's ability to scan to the impaired side by increasing head movements and fast eye movements [8]. Such adaptive treatment has been reported to improve speed and accuracy of visual search into the hemianopic side with significant improvement of functional visual behaviour [58, 59]. Web-based therapies are also freely available as compensatory visual search and scanning training (http://www.readright.ucl.ac.uk/; http://www.eyesearch.ucl.ac.uk/). Other adaptive aids included the use of typoscopes to facilitate reading and use of compensatory head posture. 

Substitutive treatment options utilise devices or modifications to change the visual field such as prisms, eye patches, and magnifiers. Treatment with prisms involves expanding the visual field in lateral gaze [60]. Prisms displace images of objects in the “blind” visual field across into the seeing side serving as a cue for the patient to make scanning head, and eye movements to the blind side to locate the objects. There is doubt as to the long-term efficacy of this treatment as many patients do not continue long-term wear of their prisms [61]. Six percent of our patients were given Peli prisms to widen their field of view optically, but none continued long-term wear of their prismatic glasses.

Visual fields can recover spontaneously following damage to the geniculostriate pathway after cerebral infarction [9]. Generally, patients with homonymous field defects from vascular disease seem to have a poor prognosis for spontaneous recovery [9]. Pambakian and Kennard [26] reported that less than 10% fully recover and up to 50% show partial improvement of varying extent. Zhang et al. [5] reported that of those diagnosed with visual field loss within one month of stroke onset, 55% showed improvement of field. Gray et al. [30] reported most recovery in the first 10 days which is supported by Cassidy et al. [49]. Further recovery is negligible after 10–12 weeks [30, 62]. Importantly, no patient, lesion, or visual field type was found to correlate with outcome [5]. Our outcome results were very similar. Eight percent achieved full recovery of their visual field loss within the first 2 weeks of stroke onset. Subsequently, a further 39% showed partial improvement with improvement occurring within 3 months of stroke onset. However, 52% showed no improvement and a very small number showed further deterioration. Our findings are also consistent with those of Zhang et al. [5] in that we found no correlates for those who improved or did not show change in visual field. There was a wide age range of patients recruited to this study (aged 1–94 years). However, the majority were aged 55 years or older with a mean of 69 years and a median of 71 years. The age range was not found to significantly impact on the final outcome for our patients.

The problems specifically caused by hemianopia include hemianopic reading deficits [11, 63], impaired visual exploration of the hemianopic side [8], and deviated subjective midline in spatial localisation [64–68]. Kerkhoff [67] reported that two-thirds of all visual field defect patients in rehabilitation have chronic impairment in visual search and reading and have visual symptoms.

Hemianopic alexia results from loss of parafoveal field area which cuts the perceptual window for reading [69]. The perceptual window extends approximately 13 letters to the right of fixation and 6 letters to the left of fixation. Thus, right hemianopia impairs detection of full words, while left hemianopia leads to missed first letters/start of sentences resulting in slow reading for right hemianopia and faulty reading for left hemianopia [70, 71]. 

Visual search problems include numerous hypometric saccades and frequent repetitions in to the hemianopic side [11, 72, 73]. Zihl [11] reported that patients spend more time attending to their good side than the hemianopic side and fail to scan or search their hemianopic side quickly enough to comprehend the full scene. Patients can have a skewed midline for spatial localisation [67], and it is possible that this shift may impact on mobility. Consequently, there is significant impact to activities of daily living with increased risk of collisions and accidents [74], and this defect is persistent long term [67]. 

The presence of persistent, complete homonymous hemianopia is reported as being associated with a poor prognosis for rehabilitation and survival [34, 75–80]. Diminished vision related quality of life is correlated with the extent of visual field loss. Gall et al. [81] found that visual field loss patients had significant reduction of vision related quality of life compared to healthy controls. Larger visual field defects were associated with more distress on health related quality of life. Furthermore, independence related to driving ability may be lost. In many countries, the standards are a horizontal field of vision of 120 degrees and no significant defect within 20 degrees of central fixation. However the large variances in driving performance versus extent of visual field loss mean than individual driving assessments are recommended regardless of the type and extent of visual field loss [82]. 

It is not uncommon for stroke survivors to have coexistent visual inattention/neglect in association with their visual field loss particularly in right-sided hemisphere stroke lesions [83]. In severe cases this can make the detection and differential diagnosis of the visual field defect very difficult, and at times it is not possible to make the diagnosis in the acute phase. It is also common for visual field loss to be associated with poor central vision and abnormal eye movements [7, 31]. We found a considerable proportion of our patients with visual field loss to have associated visual impairment which included low vision, eye movement abnormalities, and visual perceptual problems. Notably, the additional visual impairments did not appear to further significantly impair quality of life. 

Quality of life data was gathered in 63 of our patients with visual field loss. The ADLDV questionnaire was utilised which specifically measures vision-related activities of daily living for visual recognition and ability to “see and do,” impact on mobility plus near vision activities such as reading and sorting money. Our patients with a normal visual status at baseline assessment had a mean ADLDV score of 81.6 : 88 is the maximum normal score. For those with solely visual field loss, the mean score was 69.43, and for those with other associated visual impairments, the mean score was 63.55. There was no significant difference for having a sole or multiple visual impairments, but there was considerable variability and overlap of scores between groups. Multiple regression analysis did not reveal any correlation for key variables such as gender, age at onset of stroke, the length of time from stroke onset to time of first eye examination, the visual diagnosis or laterality, and type of stroke. Confidence intervals for these variables contained the zero value, and thus it is possible for the correlation to be zero. It is however recognised that there are many other variables that may affect quality of life scores that are also independent of vision. Reduction in scores was across all levels of visual recognition, mobility, and near vision tasks. It is important to maximise compensation to visual field loss to enhance visual function and related activities of daily living. The knowledge that visual field loss significantly impacts on quality of life should underpin the requirement for appropriate screening and diagnosis of visual field loss and associated visual impairments following stroke.

There are a number of limitations to this study. The VIS study only includes those patients referred with suspected visual impairment and therefore is not representative of a full stroke patient cohort. Given that a number of our patients were asymptomatic it is possible that referrals were not made for other visually asymptomatic patients. The area of stroke lesion was based on the neuroimaging reports available at the time of recruitment for our patients. These documented either the main stroke lesion area or the artery affected. Therefore we may have missed documenting other areas affected by the stroke that were not specified on the scan report. For some of our patients with visual field loss but with imaging reports stating the site of stroke lesion in the cerebellum or brainstem, we have assumed a nonreported extension of the stroke to nearby areas in which the visual pathway may have been damaged. 

Two-thirds of our assessments at baseline were by confrontation technique which we believe is reliable for detecting homonymous hemianopia and large defects but less reliable for smaller visual field defects. It is possible that the latter types of visual field loss were not detected. We were unable to obtain followup on all patients offered review appointments. This was due to patient death, moving out of area, loss to followup, or patient choice not to attend followup. As a pragmatic study, we chose to describe the outcomes of the patients who did attend followup, as data on 199 cases was felt of sufficient number to report on. However, there was variable followup of these patients from 2 weeks to 3 months, and thus it was not possible to plot consistent trajectories of visual field change over time. Furthermore there was insufficient data to enable us to plot percentage of recovery rates reliably over time. Such information would be useful in future longitudinal studies of visual fields. 

Only 63 of our patients completed quality of life questionnaires which is a very small percentage of those with visual field loss. Completion of the ADLDV questionnaire was not a mandatory assessment in our study, and given that this was an unfunded study with limited clinical resources, the questionnaire was only completed if clinicians had sufficient time to do so. The low number undoubtedly limited the extent to which we could evaluate differences between groups for absence of visual field loss versus visual field loss only or visual field loss with other visual impairments. Future prospective studies and trials reporting outcomes for stroke patients with visual field loss should aim to incorporate quality of life and activity of daily living assessments as important outcome measures. 

## 5. Conclusions

To our knowledge, this is the first large prospective cohort observation study of visual field loss in stroke survivors in the UK. 52% of our cohort had visual field loss, and 47% had visual field loss due to their recent stroke once preexisting visual field loss was accounted for. Most patients were aware of their visual field loss. However 10% were asymptomatic which may have implications to activities of daily living such as driving. Other symptoms attributed to coexistent visual impairment included diplopia and blurred vision. Visual symptoms relating to visual field loss included hallucinations and spatial awareness difficulties. Three-quarters had homonymous hemianopia. The area of stroke largely related to the type of visual field defects. Specifically, occipital, temporal, and parietal lobe strokes and middle and posterior cerebral artery strokes were linked to hemianopia, quadrantanopia, altitudinal, temporal crescent, and homonymous scotoma defects. Visual rehabilitation consisted of compensatory or substitutive options. Full resolution of visual field loss occurred for 8% of patients, partial improvement for 39%, and no recovery for 52%. Many patients had other visual impairments in addition to visual field loss. Regardless of this, vision-related activities of daily living were reduced in comparison to stroke survivors without visual impairment. 

We recommend careful visual screening of stroke survivors to accurately diagnose presence of visual field loss and any other visual impairments so that prompt treatment can be instigated to maximise visual function and outcomes for these patients.

## Figures and Tables

**Table 1:** Reasons for exclusion.

Unable to consent	Discharged without assessment	Transient ischaemic attack	Unwilling to consent	Not available for assessment	Died	Other pathology	Failed to attend assessment	No reason provided
225	52	44	34	28	26	10	4	7

**Table 2:** Types of visual field loss.

Type	Number (total: 479)	Percentage
Complete homonymous hemianopia	259	54.5
Partial homonymous hemianopia	79	16.6
Constriction	44	9.3
Inferior quadrantanopia	40	8.4
Superior quadrantanopia	30	6.4
Hemianopia and contraquadrantanopia	6	1.2
Scotoma	5	1.0
Chequerboard	3	0.6
Altitudinal	3	0.6
Complete unilateral blindness	2	0.4
Binasal hemianopia	1	0.2
Bilateral homonymous hemianopia	1	0.2
Temporal crescent	1	0.2

**Table 3:** Area affected by stroke lesion.

Area of brain (combined single and multiple sites of lesion)	Number (total: 1001)	Percentage
Occipital lobe	225	22.5
Parietal lobe	165	16.5
Middle cerebral artery	81	8.1
Cerebellum	79	7.9
Frontal lobe	74	7.4
Brainstem	69	6.9
Temporal lobe	62	6.2
Thalamus	58	5.8
Basal ganglia	56	5.6
Lacunar	35	3.5
Internal capsule	29	2.9
Posterior cerebral artery	21	2.1
Anterior circulation infarct	16	1.6
Periventricular	14	1.4
Intraventricular	7	0.7
External capsule	5	0.5
Anterior cerebral artery	4	0.4
Posterior inferior cerebellar artery	1	0.1

**Table 4:** Area of brain stroke and recorded type of visual field loss.

	Homonymous hemianopia	Homonymous quadrantanopia	Constricted loss	Scotoma	Altitudinal	Bilateral loss	Temporal crescent loss
Frontal lobe	5	2	1				
Parietal lobe	20	6	6				
Temporal lobe	1						
Occipital lobe	78	1	23	2	1	2	
Brainstem	4		3				
Cerebellum	4	2	2				
Basal ganglia	7		2				
Thalamus	2	1	2				
Internal capsule	6						
Periventricular	4						
Intraventricular	3	1					
Lacunar	4	2	1				
Anterior cerebral artery	2		1				
Middle cerebral artery	28	6	2				
Posterior cerebral artery	15	4	1				
Anterior circulation infarct	2	2	1				
Multiple brain areas	158	18	16	2	2	5	1

**Table 5:** Visual rehabilitation.

Treatment options								
	Refraction	Peli prisms	Diplopia prisms	Occlusion	Low visual aids	Typoscope	Orthoptic exercises	Advice
Sole treatment option (*n* = 288)	22	5	3	5	2	0	0	250
Multiple treatment option (*n* = 186)	63	24	10	2	18	42	8	224

Advice consisted of raising awareness of visual field loss, reading strategies, scanning eye and head movements, use of lighting, compensatory head posture, and registration for visual impairment.
